# Frag-Virus: a new term to distinguish presumptive viruses known primarily from sequence data

**DOI:** 10.1186/1743-422X-5-34

**Published:** 2008-02-27

**Authors:** Alexander Voevodin, Preston A Marx

**Affiliations:** 1 Vir&Gen, Toronto, Canada; 2 Division of Microbiology, Tulane National Primate Research Center, Covington, Louisiana USA

The wide availability of PCR-based methods for the amplification of nucleic acids homologous to known viral genomes plus automated DNA sequencing have led to explosive growth of a new field, 'proxy' virus isolation. Typically, genomic fragments are amplified and subjected to phylogenetic analysis showing that they are related to, but distinct from, known '*bona fide*' viruses. These "new viruses" are now commonly reported in the virological literature and are too numerous to cite here, two studies are given as typical examples [1,2]. Although unintentional, these reports may, mislead the readership of scientific journals and the general press. Having no distinction between preliminary genome-based evidence and conclusive proof by biological isolation and characterization of a replication-competent virus blurs the meaning of new virus.

To distinguish presumptive viruses known primarily through genomic sequence fragments from *bona fide *viruses we propose the term **'frag-virus'**.

The 'frag-virus status' may be intermediary between 'full' recognition as a new virus, as occurred, for example, with hepatitis C virus and Kaposi sarcoma herpesvirus [3-6]. Frag-virus designation may also be a category for such virus that likely exists, but there is neither sufficient motivation nor resources to pursue its elevation from frag-virus limbo to *bona fide *virus status. At the same time the frag-virus status will be a 'career end-point' for sequences belonging to non-infectious 'pseudo-viruses' or those having their origins in artifact.

Whatever the 'fate' of frag-viruses, this simple term is highly informative and may prove useful in preventing misconceptions about new viruses.
